# Analysis of CAUTIs and Projected Effect of Increasing Pyuria Threshold in Urinalysis with Reflex to Culture

**DOI:** 10.1017/ash.2024.196

**Published:** 2024-09-16

**Authors:** Kristen Simpson, Nora Colburn, Brandi Manning, Sydney Agnello, Shandra Day

**Affiliations:** The Ohio State University

## Abstract

**Background:** Catheter-Associated Urinary Tract Infections (CAUTI) are preventable hospital acquired infections that contributes to patient morbidity, prolonged hospital stays and increased healthcare costs. Complete compliance to the CAUTI bundle is critical for preventing infection—not only reducing catheter days, but also ensuring appropriate indications are present for urine culture collection. **Methods:** This retrospective study included 145 patients diagnosed with CAUTI per NHSN definitions from July 1, 2020 to June 30, 2023. Data collected included laboratory data, catheter duration, catheter indication, urinalysis/culture indication and if foley was appropriately removed/changed prior to specimen collection. A urinary catheter indication order was implemented in February 2021 requiring providers to select specific criteria for catheter placement/maintenance. In July 2023, the threshold for urinalysis to reflex to culture was increased to ≥10 WBCs and this criteria was applied to these cases to estimate the effect on diagnosis of CAUTI. **Results:** The most common indications for urinary catheters were input and output monitoring 76 (52%) and urinary retention/obstruction 34 (23%). No indication was entered on 22 (15%) patients. No difference was seen in the number of catheters without an indication before or after the 2021 order update. Mean catheter duration was 11.5 days with a median of 7 days. The most common indications for obtaining a urine specimen were leukocytosis/fever/sepsis 91 (63%), urinary symptoms/abdominal/flank pain 13 (9%), urine appearance 6 (4%), and altered mental status 4 (3%). In 31 (21%) patients, no indication was identified. A urinalysis with reflex to culture was completed in 105 (72%) and the catheter was removed prior to culture collection in 68 (47%). Of the 127 patients with a urinalysis and culture, 11 had 0-5 WBCs, 16 had 6-9 WBCs, 15 had 10-20 WBCs and 85 had >20 WBCs. Using the new pyuria criteria for urinalysis to reflex to culture, 27 (19%) CAUTIs could have been avoided. **Conclusion:** Review of CAUTI cases identified opportunities for improving documentation and education of appropriate indications for urinary catheters and evaluation of urinary tract infection. The majority of urine cultures were obtained due to non-specific symptoms and less than 10% had specific urinary symptoms indicating need for continued education and diagnostic stewardship. Increasing the pyuria threshold needed to reflex to culture has the potential to significantly reduce CAUTIs but additional education is needed to ensure catheters are changed prior culture collection and specimens are only sent when signs and symptoms of urinary tract infection are present.

